# Human Schwann Cells Seeded on a Novel Collagen-Based Microstructured Nerve Guide Survive, Proliferate, and Modify Neurite Outgrowth

**DOI:** 10.1155/2014/493823

**Published:** 2014-05-11

**Authors:** Sabien G. A. van Neerven, Laura Krings, Kirsten Haastert-Talini, Michael Vogt, René H. Tolba, Gary Brook, Norbert Pallua, Ahmet Bozkurt

**Affiliations:** ^1^Department of Plastic Surgery, Reconstructive Surgery, Hand Surgery and Burn Injuries, RWTH Aachen University Hospital, Pauwelsstraße 30, 52074 Aachen, Germany; ^2^Institute of Neuroanatomy, Hannover Medical School, Carl-Neuberg-Straße 1, 30625 Hannover, Germany; ^3^Center for Systems Neuroscience (ZSN), Carl-Neuberg-Straße 1, 30625 Hannover, Germany; ^4^Core Facility Two-Photon Microscopy, RWTH Aachen University Hospital, Pauwelsstraße 30, 52074 Aachen, Germany; ^5^Department of Laboratory Animal Sciences, RWTH-Aachen University Hospital, Pauwelsstraße 30, 52074 Aachen, Germany; ^6^Institute of Neuropathology, Medical Faculty, RWTH Aachen University, Pauwelsstraße 30, 52074 Aachen, Germany; ^7^Jülich-Aachen Research Alliance (JARA), Translational Brain Medicine, 52425 Jülich, Germany

## Abstract

A variety of new bioartificial nerve guides have been tested preclinically for their safety and nerve regeneration supporting properties. So far, only a limited number of biomaterials have been tested in humans since the step from preclinical work to a clinical application is challenging. We here present an *in vitro* model with human Schwann cells (hSCs) as an intermediate step towards clinical application of the nerve guide Perimaix, a collagen-based microstructured 3D scaffold containing numerous longitudinal guidance channels for directed axonal growth. hSCs were seeded onto different prototypes of Perimaix and cultivated for 14 days. hSC adhered to the scaffold, proliferated, and demonstrated healthy Schwann cell morphology (spindle shaped cell bodies, bipolar oriented processes) not only at the surface of the material, but also in the deeper layers of the scaffold. The general well-being of the cells was quantitatively confirmed by low levels of lactate dehydrogenase release into the culture medium. Moreover, conditioned medium of hSCs that were cultivated on Perimaix was able to modify neurite outgrowth from sensory dorsal root ganglion neurons. Overall these data indicate that Perimaix is able to provide a matrix that can promote the attachment and supports process extension, migration, and proliferation of hSC.

## 1. Introduction

Peripheral nerve injuries (PNI) can lead to the permanent loss of motor, sensory, or autonomic function [1, 2] and repair strategies often appear insufficient. Currently autologous nerve transplantation (ANT) is regarded as the gold standard in the repair of severe PNI. Yet harvesting these nerves is obviously associated with comorbidities at the donor site and the source of donor nerves is only limited. As a consequence, much effort has been invested in the preclinical testing of novel biomaterials intended as nerve guidance structures that can serve as an alternative to ANT. A key requirement is that these biodegradable nerve guides have to be cytocompatible (i.e., non-toxic, enabling cell adhesion, promoting migration, and proliferation) with cells and axons resident in the microenvironment of the peripheral nerve [3–6]. Schwann cells (SC) are, under normal physiological conditions, in intimate contact with axons and are responsible for myelin production and ensheathment of the axon. Their prominent role becomes even more evident in response to PNI, where these cells not only create an axonal growth permissive environment by the production of trophic factors, but also direct and provide physical support to regrowing axons by creating “Bands of Büngner.” These SC can be either endogenously present, migrating from the proximal and distal nerve stumps, or exogenously derived by functionalizing nerve implants with SC. Regardless of their origin, materials intended as nerve guidance structures need to be cytocompatible with regrowing axons and SC [3–6]. Previous* in vitro* studies from our group demonstrated the cytocompatibility and growth supporting properties of the collagen-based microstructured 3D nerve guide, Perimaix, with rat Schwann cells (rSC) [3, 4]. Viable rSC did not only adhere to the nerve guide, but also migrated throughout the guidance channels. Of particular importance was the observation that rSC formed cellular columns within the guidance channels reminiscent of “Bands of Büngner,” which are crucial structures for successful peripheral nerve regeneration and that are formed during the natural process of Wallerian degeneration [4]. Furthermore axonal guidance by Perimaix was demonstrated by explanting rat dorsal root ganglia (DRG) onto the collagen scaffolds; SC resident in the DRG migrated into the longitudinal oriented channels of Perimaix, adhered to the collagen, and provided a physical support to outgrowing neurites [3]. Moreover general cytotoxicity tests confirmed the biocompatibility of Perimaix with rSC [3, 4].

Nevertheless, envisioning a clinical application for Perimaix requires the critical step of verifying its cytocompatibility with cells of human origin. Hence, we investigated whether human Schwann cells (hSCs) were able to adhere, proliferate, and migrate on different prototypes of Perimaix. Additionally, the axonal growth supportive properties of these cells were investigated by obtaining conditioned medium from the Perimaix-adherent hSC and demonstrating its growth promoting effects on rat DRG neurons.

Our data demonstrate that hSCs adhere to and grow on the collagen material. The adherent hSCs on Perimaix were viable demonstrated by expression of the Schwann cell markers vimentin and S100*β*, and cells adopted a healthy Schwann cell morphology (i.e., spindle shaped cell bodies with bipolar processes). Lactate dehydrogenase release of hSC seeded on Perimaix was very low and comparable to normal culture conditions, indicative of the general well-being of the cells on the scaffold.

## 2. Material and Methods

### 2.1. Isolation and Purification of Human Schwann Cells (hSCs)

Schwann cells from human peripheral nerves were kindly provided by Haastert et al. (Department of Neuroanatomy, Hannover Medical School, Germany) with permission of their local ethical review committee. HSCs were isolated and highly enriched under serum-free conditions [7] and further cultivated according to an established protocol, which was previously used for the cultivation of rat Schwann cells (rSC) [4]. As such, for propagation of hSC cultures, medium consisted of advanced Dulbecco's Modified Eagle's Medium (DMEM: Gibco, 21969-035), 10% fetal calf serum (FCS) gold (PAA, 49/A15-151), 1% Glutamax, and 0.1% gentamicin (Sigma-Aldrich, G1397) supplemented with growth factors: 0.0417 ng/mL human basic Fibroblast Growth Factor (bFGF) (PeproTech, 100-18b), 0.0417 ng/mL human heregulin (PeproTech, 100-03), 0.475 ng/mL Forskolin (Sigma-Aldrich, F6886), 0.1% gentamicin (Sigma-Aldrich, G1397), and 1% Glutamax. Plates and culture flasks were always coated with poly-L-lysine (100 *μ*g/mL, Sigma-Aldrich P2636, overnight at 37°C)/laminin (50 *μ*g/mL, Sigma-Aldrich). After propagation in serum, hSC cultures needed additional purification via p75NGFR antibody labeling (AHU0302, Invitrogen) followed by magnetic bead cell separation (MACS, Miltenyi Biotec). This additional purification method again yielded Schwann cell cultures of approximately 98% purity.

### 2.2. Preparation of Perimaix Prototypes

Perimaix was provided by Matricel GmbH (Herzogenrath, Germany). The preparation of these nerve guides with longitudinal pore channels (patented “unidirectional freezing” process followed by freeze-drying) has been described previously [3, 4]. Briefly, collagen was isolated from porcine tissues harvested under veterinary supervision from animals declared “fit for human consumption.” The prepared collagen solution, containing 10% to 15% (w/w) elastin, was used to prepare a 1.5 wt% aqueous dispersion. The dispersion was cooled and frozen using a defined temperature gradient and a constant cooling rate. Longitudinal micropores were created via directed ice crystal growth across the collagen material induced by constitutional supercooling with appropriate freezing parameters (see also [3]).

Sublimation of the finger-shaped ice crystals during the freeze-drying process generated the dried, longitudinally orientated pores. As such, the ice crystal morphology in the frozen dispersion determines the pore structure. The pore size can be adjusted between 20 *μ*m and 100 *μ*m. Details on techniques to vary pore size were described previously [3]. The* in vitro* and* in vivo* stability of the material were tailored using a non-toxic cross-linking method based on the use of 1-ethyl-3(3-dimethylaminopropyl) carbodiimide and samples were sterilized using gamma irradiation (25 kGy). Scaffolds were provided with different modifications and were characterized by the manufacturer as described in Table 1.

### 2.3. Scaffold Seeding

hSCs were harvested by standard trypsinization and 500,000 cells were seeded on each variant of the collagen scaffolds (dimensions cylinder: 5 mm length × 2 mm diameter) in a total volume of 25 *μ*L [20,000 cells/*μ*L]. Cells were kept in culture for 3, 7, and 14 days with every second day medium renewal. Conditioned medium derived from scaffold-adherent hSC was collected at every medium change and immediately frozen at −80°C.

### 2.4. Dorsal Root Ganglia (DRG) Isolation

DRGs were isolated from the spinal dorsal column of donor adult female LEWIS rats as previously described [8]. Briefly, following isoflurane anesthesia rats were quickly decapitated and the vertebral column was exposed by the removal of overlying skin and muscles. Under a dissection microscope, the vertebral column was opened and DRGs were dissected and collected in ice cold HBSS (Hank's Balanced Salt Solution: Invitrogen 14170-088). Subsequently, spinal nerves were removed and DRGs were transferred into a HBSS filled falcon tube. Chemical dissociation of DRGs was performed under sterile culture conditions by incubation in collagenase type 1 (0.8% Collagenase type 1, Biochrom, C1–22) in collagenase buffer (100 mM HEPES, 120 mM NaCl, 50 mM KCl, 1 mM CaCl_2_, 5 mM glucose, 3% BSA, pH 7.4) for 2 hrs followed by 30′ 0.25% Trypsine treatment at 37°C. DRGs were mechanically dissociated and separated from debris and Schwann cells by centrifugation through a Percoll density gradient (1.3 mL Percoll: 450 *μ*L 0.1 M PBS: 3.2 mL H_2_O) at 1200 g (rcf) for 12 min. Neurons were resuspended in DRG medium (Neurobasal A (Gibco, 21103-049), 2% B27 supplement (Gibco, 17504-044), 1% Glutamax, 1% pen/strep) and seeded onto poly-L-lysine/laminin-coated plates at a density of 400–500 neurons per well in 24-well-plates. DRG sensory neurons were incubated with conditioned media derived from hSCs cultivated on Perimaix diluted 1 : 2 with DRG medium. The negative control (nC) condition received DRG medium combined with normal SC medium. The positive control condition consisted of 1 : 2 dilution of medium derived from hSC grown in normal culture plates (pC). Sensory neurons were then incubated with the conditioned media at 37°C, in humidified atmosphere with 5% carbon dioxide for 2 days.

### 2.5. Scanning Electron Microscopy

After* in vitro* cultivation, SC seeded scaffolds were processed for scanning electron microscopy (SEM). As such, the scaffolds were fixed for 24 h in 4% glutaraldehyde in 0.1 M PBS. After dehydration in acetone using a Polaron E3000 critical point dryer (Polaron Equipment Ltd., Watford, UK), the specimens were mounted on stubs and sputter was coated with gold, loaded into an ESEM XL30 FEG scanning electron microscope (Philips EO, Eindhoven, NL), and viewed under an accelerating voltage of 5 kV. Surface analysis after hSC seeding was done by scanning of the complete scaffolds, whereas analysis of hSCs that migrated into the microchannels was performed after dividing the scaffolds in halves.

### 2.6. Immunocytochemistry

Additionally, hSC-loaded scaffolds were used for cell specific immunocytochemical stainings. Therefore scaffolds were fixed for 30′ in 4% paraformaldehyde solution in 0.1 M phosphate buffer (PB, 4°C) and subsequently cut in 100 *μ*m sections on a vibratome (Leica VT1000S3) and mounted on glass slides. Then sections were washed 3x with 0.1 M PBS and blocked with 3% normal goat serum (Sigma, Munich, Germany), 0.5% bovine serum albumin (BSA, fraction V), and 1% Triton X-100 in 0.1 M PBS for 60 min. Sections were then incubated overnight using the following primary antibodies: mouse monoclonal antineurofilament 200 kDa (NF200; clone NE14, 1 : 5000, Sigma), mouse monoclonal antivimentin (clone V9, 1 : 20,000, Sigma), or rabbit polyclonal anti-S100*β* (1 : 1000, Dako, Hamburg, Germany). Subsequently, fluorescent conjugated secondary antibodies (goat anti-rabbit, Alexa-488, and goat anti-mouse, Alexa-594, both 1 : 500, Molecular Probes, Paisley, UK) were incubated with primary antibodies 3 h at room temperature. Subsequently every consecutive section was counterstained using the nuclear dye 40,6-diamidino-2-phenylindole (DAPI, 1 mg/mL, 5 min). Negative controls were performed via omitting primary antibodies and revealed no non-specific immunostaining.

### 2.7. Two-Photon Laser Scanning Microscopy (TPLSM)

Sections were analyzed using a pulsed Ti-Sapphire laser (MaiTai DeepSee, SpectraPhysics) attached to an upright two-photon microscope system (FV1000MPE, Olympus Corp., Tokyo, Japan). A 25x water immersion objective was used for imaging (1.05NA, WD2.0). For fluorescence excitation of Alexa 488 and Alexa 594 the laser was tuned to the wavelength of 860 nm. The collagen matrix was visualized by the non-linear optical effect of second harmonic generation (SHG) at the same wavelength. The emission signals of Alexa 488, Alexa 594, and SHG were collected with bandpass filters at 505–525 nm, 560–660 nm, and 430–470 nm, respectively. Series of subsequent *xy*-frames with 1024 × 1024 pixels in 1 *μ*m *z*-steps were obtained for structural 3D reconstruction of the sample. A *z*-projection of the image stacks using the software FluoView ASW (Olympus) and merge of channels was used to define hSC morphology and alignment along the microporous channels. Image analysis was conducted with Imaris Software (Bitplane, Zürich, Switzerland). Contrast enhancement was performed using Adobe Photoshop 7 software (Adobe Systems, Munich, Germany).

### 2.8. Cell Toxicity: Lactate Dehydrogenase (LDH) Release

In addition to immunocytochemical observations, described below, cytotoxicity of treatment conditions was measured using lactate dehydrogenase (LDH) release into the medium (Roche Cytotoxicity Detection Kit). LDH release was normalized with respect to maximal LDH release after total cell lysis [9, 10].

### 2.9. Statistical Analysis

All data are represented as means with +/− standard error of the mean (SEM). All statistical analyses were performed using Graphpad Prism Software (San Diego, USA). After passing normality test, One Way ANOVA with Bonferroni posttest was performed. A *P* value of 0.05 was considered as the level of statistical significance.

## 3. Results

### 3.1. Perimaix

**Figure 1:**
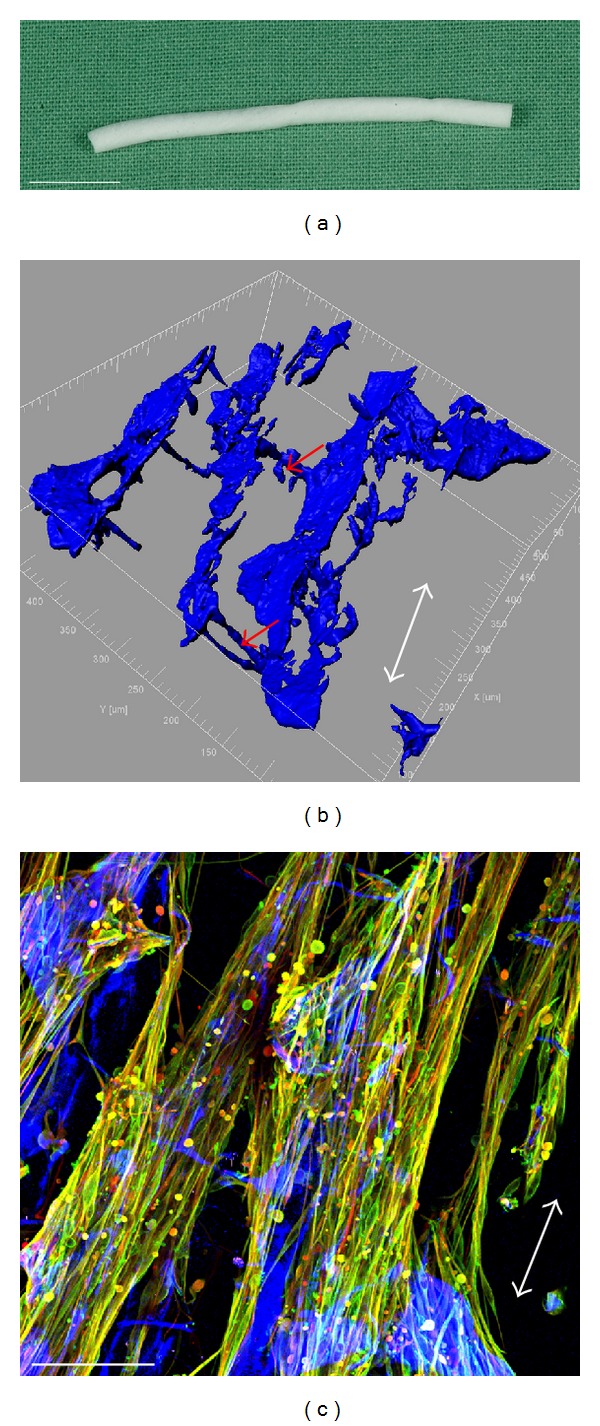
Macroscopic and TPLSM images of Perimaix. (a) Macroscopic photograph (scale bar 10 mm). (b) TPLSM 3D computer processing after visualisation of the collagen structure via SHG at 860 nm (the collagen structure was artificially coloured blue by the computer software, scale bar 100 *μ*m). Note the collagen interconnections (red arrows) providing structural support to the material and preventing collapsing of the microchannels. (c) Original TPLSM image after SHG and immunocytochemistry (white arrows indicate channel-orientation).

Figure 1 demonstrates the nerve guide Perimaix, which comprises porcine collagen type 1 and is manufactured with longitudinal oriented and continuous microchannels for directed axonal growth.  Figure 1(a) shows a macroscopic image of the scaffold, whereas Figures 1(b) and 1(c) demonstrate TPSLM images after SHG. Note the clear orientation of the longitudinal microchannels (white arrows) and interconnections in the material (red arrows) that provide mechanical support and prevent the microchannels from collapsing.

### 3.2. Human Schwann Cell Purification

**Figure 2:**
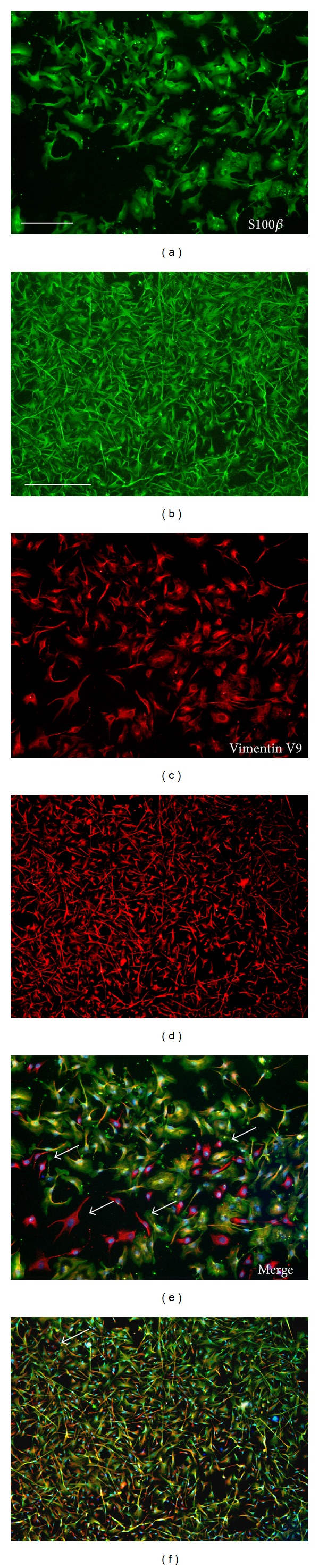
HSC cultures after propagation in serum-containing medium contained approximately 60% Schwann cells (S100*β*/vimentin positive) and 40% fibroblasts (S100*β* negative/vimentin positive (scale bar 200 *μ*m), white arrows. (a) vimentin. (c) S100*β*. (e) Merge with DAPI nuclear staining. MACS purification method resulted in Schwann cell cultures of approximately >98% purity (b), (d), and (f), scale bar 500 *μ*m. Cell purity was maintained along the culture period as well as after cell seeding onto the collagen scaffolds.

**Figure 3:**
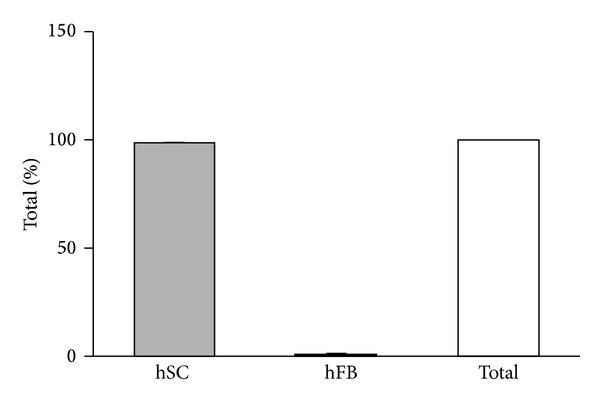
Quantification of cell purity. After MACS procedure, cultures revealed only occasionally fibroblasts (<2%) of the total amount of cells counted.

Human Schwann cells (hSCs) were derived from human mixed (sensory and motor) donor nerves. Highly enriched cell cultures [7] were isolated under serum-free conditions and propagated in serum-containing medium, so to warrant cell purity cells that were additionally purified according to MACS procedure. After propagation in serum-containing medium (prior to MACS procedure) the cultures contained approximately 60% S100*β* positive and vimentin positive hSC and approximately 40% vimentin positive/S100*β* negative fibroblasts (Figure 2). After MACS procedure, the p75NGF positive cell fraction was positive for S100*β* and vimentin. Cell counting showed a highly purified hSC culture of approximately 98 +/− 1% hSC and 2 +/− 0.5% fibroblasts (Figure 3, quantification of cell purity after additional MACS procedure). This level of cell purity was maintained and monitored until cells were seeded onto the scaffolds (Figures 2 and 3). White arrows demonstrate vimentin positive/S100*β* negative fibroblasts.

### 3.3. Cell Seeding and Cell Toxicity

**Figure 4:**
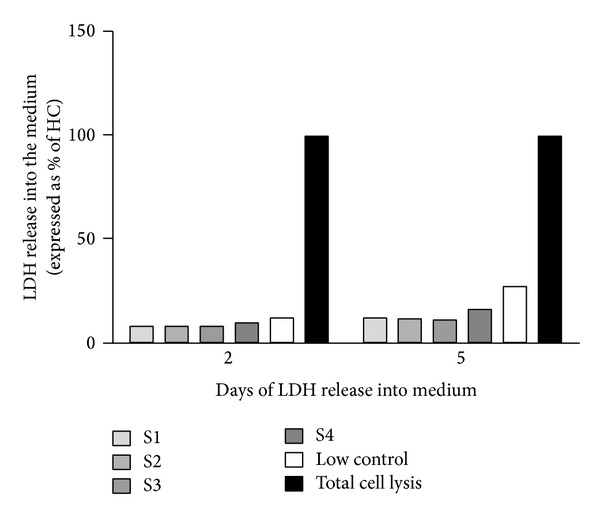
Human Schwann cell viability seeded on Perimaix prototypes S1–4: LDH release into the medium was measured at 2 and 5 days in culture (until medium change occurred). All investigated prototypes of Perimaix demonstrated LDH levels similar to the low control condition (cells in normal culture plate without any treatment).

After seeding onto the different Perimaix prototypes, LDH release into the culture medium was assessed over a period of 14 days hSC resided on Perimaix until medium change occurred (2 and 5 days). Hence, maximally five days of uninterrupted LDH release into the medium revealed very low levels of LDH (in samples 1–4; 11.97%; 11.51%; 10.76%; 16.07%, resp.) when compared to maximal LDH release (total cell lysis, Figure 4). Furthermore LDH levels were in the range of normal LDH release under standard culture conditions (27.03%), indicative of the general well-being of the cells on Perimaix.

### 3.4. Cell Seeding and Morphology

At the surface of the scaffold, hSCs were abundant and covered the walls of the microchannels. hSC formed bundles and clusters of cells that attached to the collagen material. Moreover cells displayed a healthy morphology (i.e., spindle shaped cell bodies with long bipolar projections), oriented along the microchannels, and extended their processes.

**Figure 5:**
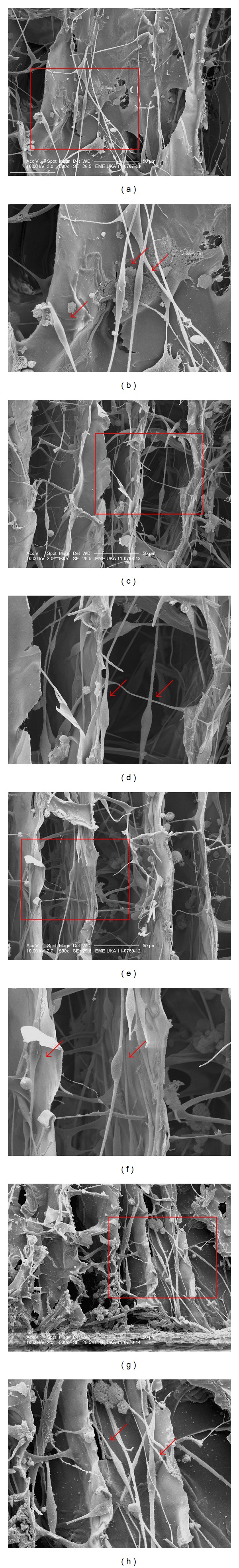
SEM images of hSCs seeded onto the different Perimaix prototypes for 14 days ((a), (c), (e), and (g) are S1–4, respectively, images (b), (d), (f) and (g) are magnifications of the rectangles in (a), (c), (e) and (g). Scale bars in (a), (c), (e) and (g): 50 *μ*m). Morphology and Schwann cell behaviour was qualitatively analysed in the inner structures (center) of the different scaffold prototypes 14 days after seeding, revealing spindle shaped cells that extended bipolar processes along the microchannels.

Scaffolds were cut in half along the axis to examine the deeper layers of the scaffold (Figure 5). hSCs were found in the center of the scaffold, demonstrating a healthy morphology and displaying attachment to the collagen walls (Figure 5, red arrows). However, occasional cells appeared to be suspended with the lumen of the micropores, probably straddling contact points on adjacent facing walls. No difference in adherence, cell morphology, or LDH release could be observed between the different Perimaix prototypes.

**Figure 6:**
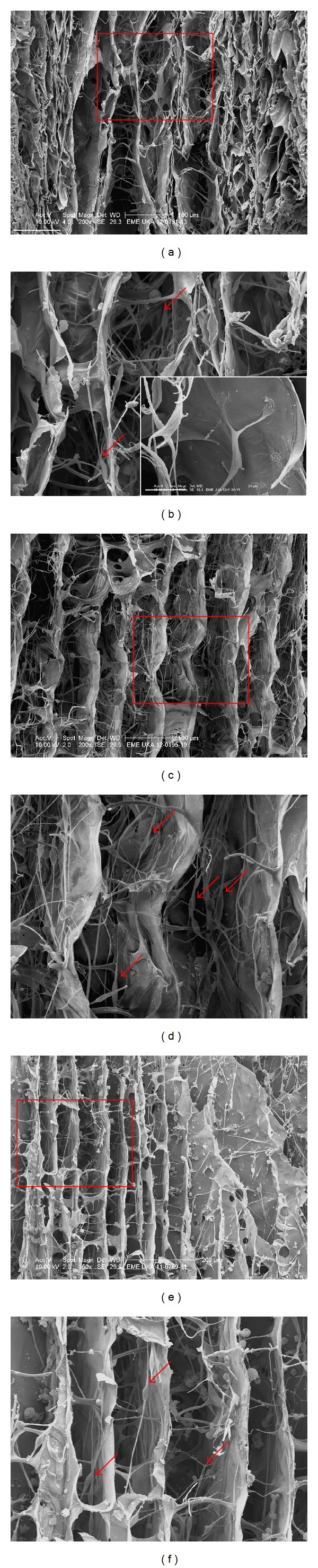
SEM images of hSC seeded on Perimaix S3 in time. hSCs were already visible in the microchannels in the center of the scaffold at 3 days ((a), (b)) after seeding, where cells adhered (magnification, scale bar 20 *μ*m) to the collagen material. hSCs remained in the scaffold and were detectable up to 7 ((c), (d)) and 14 days after seeding ((e), (f): images (b), (d), (f) and (g) are magnifications of the rectangles in (a), (c) and (e). Scale bars in (a), (c), (e): 100 *μ*m).

Cells were analysed at different time points after cell seeding (Figure 6). Three days after seeding hSCs were already detectable in the inner parts of the scaffold (Figures 6(a) and 6(b)) and adhering to the collagen walls. One week after seeding, cells were abundant and spreading into the microchannels, orientating along the collagen structure and adopting a spindle shaped morphology with long slender processes extending in bipolar direction (red arrows). Moreover, after two weeks hSCs were still present in the center of the scaffold (Figures 6(e) and 6(f) magnifications).

**Figure 7:**
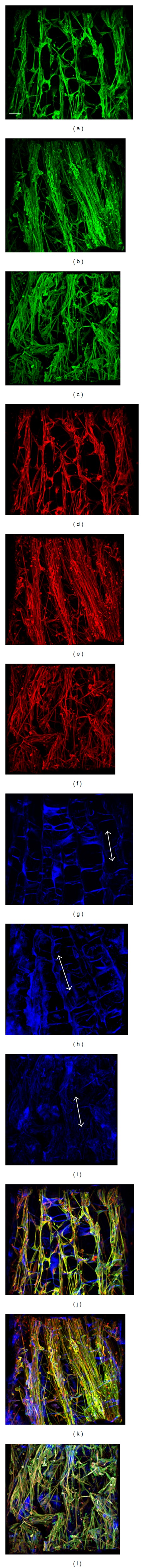
TPSLM images of hSCs seeded onto Perimaix S2 prototypes at 3 ((a), (d), (g), and (j)), 7 ((b), (e), (h), and (k)), and 14 ((c), (f), (i), and (l)) days after seeding. (a), (b), and (c): S100*β*; (d), (e), and (f): vimentin; (g), (h), and (i): collagen structure visualized by SHG; (j), (k). (l) Merge, scale bar 50 *μ*m. Surface analysis of the scaffolds demonstrated many hSCs that clustered in bundles orienting along the microchannels. Note in blue the clear length orientation (channel-orientation indicated by the white arrow) of the microchannels, with collagen interconnections to provide stability.

Figure 7 demonstrates immunohistochemically the clustering of multiple aligned cells and processes (particularly evident in Figures 7(b), 7(e), and 7(h)) forming Bands of Büngner-like profiles within the scaffolds microchannels. Cells and processes expressed both markers S100*β* and vimentin, confirming the maintained purity of the cell population and also indicating that, under the present conditions, the small fibroblast contamination was unable to expand to the extent that it could compete with the hSC for space within the microchannels.

HSCs were found abundantly on the surface of the scaffold already after 3 days after seeding, clustering in bundles and extending their processes along the microchannels of the collagen material (Figures 7(a), 7(d), 7(g), and 7(j)). By 7 days and 14 days after seeding, hSCs were abundant and covered the complete surface of the scaffold.

**Figure 8:**
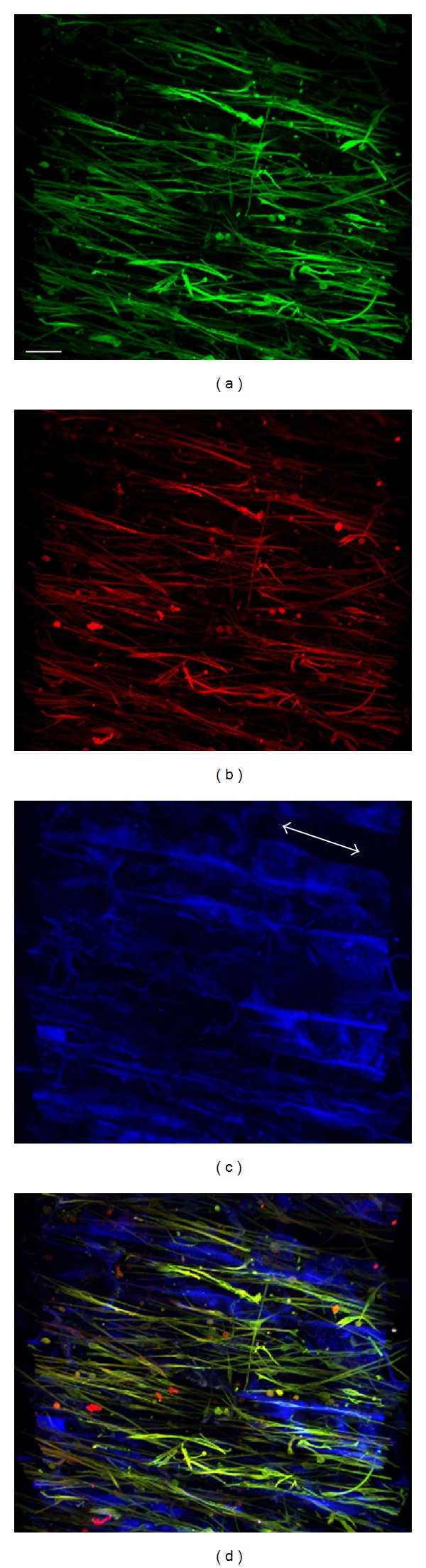
TPSLM images of hSCs in the center of the scaffold 14 days after seeding. (a) S100*β*, (b) vimentin. (c) Collagen structure visualized by SHG. (d) Merge. This example of Perimaix prototype S1 served as a growth permissive niche for hSCs; cells expressed the classical SC markers vimentin and S100*β*, adhered to the collagen walls as well as to each other, and oriented along the microchannels (channel-orientation indicated by the white arrow), adopting a bipolar morphology typical for SCs. Scale bar 50 *μ*m.

Thick sections (100 *μ*m) were made on a vibratome to investigate the deeper layers of the scaffold. Also in the middle of the scaffold cells adhered to the collagen and expressed both vimentin and S100*β* (Figure 8).

Cells elongated along the microchannels and adhered to the collagen material. They demonstrated normal Schwann cell phenotype, that is, spindle shaped cell bodies with slender processes extending along the microchannels and expressing the markers vimentin and S100*β*.

### 3.5. Neurite Outgrowth Assay

To verify whether hSC seeded on Perimaix released factors that are supportive to neurite outgrowth, conditioned medium was collected from hSC seeded on Perimaix and administered to primary sensory neurons isolated from rat DRG.

**Figure 9:**
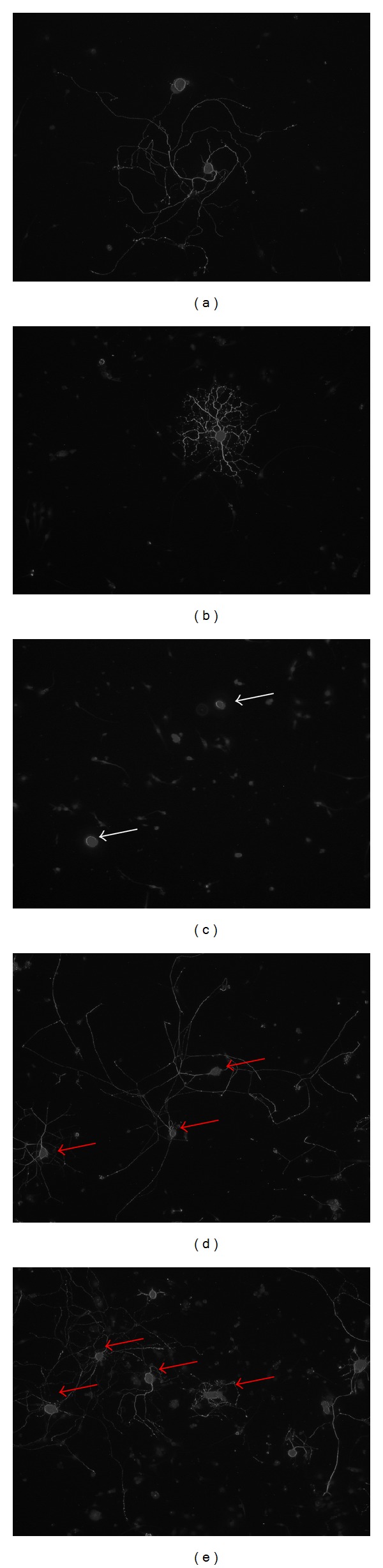
Neurite outgrowth of primary DRG sensory neurons treated with conditioned medium of hSC cultivated on Perimaix. ((a), (b)) In culture DRG sensory neurons adopt primarily two phenotypes: elongated (a) or arborised (b) [11]. (c) Normal culture medium (negative control, nC) did not induce neurite outgrowth. (e) hSCs cultivated on Perimaix released soluble factors that induced neurite outgrowth in rat DRG neurons to a similar extent as when neurons were treated with conditioned medium derived from hSC grown in a normal culture plate, (positive control pC, (d)).

Figures 9(a) and 9(b) demonstrate the different phenotypes primary sensory neurons generally adopted in culture. When DRG sensory neurons received normal (unconditioned) hSC culture medium for 48 hrs they extended no neurites (negative control, nC Figure 9(c)). In contrast, when these sensory neurons received conditioned medium from hSC cultivated on Perimaix for two days, they extended long neurites (Figure 9(e)). This neurite outgrowth was of a similar extent as when DRG neurons received medium from hSC cultivated in a normal culture plate (positive control, pC, Figure 9(d)).

## 4. Discussion

Currently the autologous nerve transplantation (ANT) is still regarded as the gold standard for the reconstruction of severe nerve injuries in humans. Despite the fact that during the last decades many biomaterials have been successfully tested and evaluated preclinically as an alternative to the ANT, relatively few new conduits or scaffolds have been studied in the clinical domain yet. This may be the result of several causes, prominent by the fact that human nerve injuries are heterogeneous in location and severity and are obviously not as standardized as animal models [5]. This may have contributed to the fact that there are, at present, only a small number of conduits that have been approved by the FDA and the EU for use for bridging short gaps (less than 30 mm) in lesioned human peripheral nerves [12, 13]. Nonetheless, the steady rise in the number of alternative conduits and scaffolds made from synthetic and/or natural polymers and with different structures and topographies dictates that at some point the critical step of evaluating biomaterials intended as nerve guides for human use must be made. In this respect we suggest that evaluating nerve guides with cells of human origin* in vitro* is a logical intermediate stage in this development process.

We have therefore seeded Perimaix, a collagen-based nerve guide, with Schwann cells from human origin and evaluated its biocompatibility. Originally, the nerve guide was developed as a substitute for the natural perineurium of the nerve. With its longitudinal oriented microchannels, Perimaix provides mechanical support to regenerating axons [3, 6] and provides a protective niche for nerve resident cells (e.g., Schwann cells, perineurial cells, and endothelial cells) that can migrate from the nerve stumps into the scaffold to support axonal regeneration. As the scaffold is made of collagen type 1, integral structural component of the nerve's connective tissue, the construct itself can also provide the molecular attachment and guidance cues required by migrating Schwann cells and regenerating axons. The nerve guide was already evaluated for its biocompatibility with cells from rat origin in previous studies of our group [3, 4]. Moreover,* in vivo* studies revealed excellent host tissue integration through the nerve guide bridging 20 mm nerve gaps after a regeneration period of 6 weeks and 12 weeks [6]. These promising results obtained in the sciatic nerve model prompted us to evaluate the scaffold with Schwann cells from human origin as a vital step towards clinical application.

Human Schwann cells seeded on Perimaix displayed a healthy and dedifferentiated phenotype (i.e., spindle shaped cell bodies and slender bipolar processes extending in bipolar direction) as observed by SEM and expressed the classical Schwann-cell markers vimentin and S100*β*. The orientation of the Schwann cell processes generally adopted the orientation of the microchannels of the nerve guide. The adoption of the unipolar and bipolar spindle shape by the seeded Schwann cell is reminiscent of the range of morphologies demonstrated by Schwann cells during migratory activity in simple 2D tissue culture [14].

This supports the notion of the seeded human Schwann cells migrating along the longitudinal axis of the scaffold microchannels. The same morphology was adopted by rat olfactory ensheathing cells migrating through a similarly orientated microporous collagen scaffold [15].

This healthy phenotype of the seeded hSC was confirmed quantitatively by acute cytotoxicity tests which showed no indications of excessive cell death throughout the 14-day cultivation time. More importantly, hSCs were also found in the deeper layers of the scaffold and even in the center of the conduit. As such, these data suggest a high permeability of the material to nutritional factors that are crucial for cell survival and growth. Therefore the structural concept presented here already meets one of the vital criteria for nerve guides intended for human application; it provides a protective and beneficial niche for migrating host cells.

In the framework of a supportive scaffold polymer, no difference in cell behavior could be observed between the different Perimaix prototypes, suggesting that a higher collagen content as nor the collagen content or the crosslinking degree had influence on cell adherence or growth. Probably the presence of extracellular matrix molecules (ECM), such as collagen in general, is more decisive for proper cell growth and adherence. Other studies already showed that the presence of ECM can trigger Schwann cells to produce beneficial factors to axonal growth [16]. Moreover Schwann cells themselves are also able to produce ECM molecules fibronectin, collagens [17–19], and laminins [20, 21] creating a basal lamina that makes part of the microenvironment of the peripheral nerve (endoneurium) [22, 23]. The basal lamina is essential in the Schwann cell-axon interaction and during peripheral nerve regeneration it is particularly this nerve element that is followed by the Schwann cells (creating Bands of Büngner) guiding regenerating axons [24, 25]. It is for this reason that new innovative tissue engineering approaches aim for utilizing Schwann cells to build up the scaffold matrix or that the basal lamina produced by nerve resident cells is preserved [26–28].

So as our scaffold provides a proper matrix for the hSC and we observed elongation and adherence of the cells along the collagen microchannels, this may also suggest that ECM are produced by the hSC. Amstrong and others already demonstrated that it was not the ECM molecules that influenced neurite outgrowth directly, but it was particularly the SCs that served as an intermediated cellular step responding to ECM molecules by producing factors beneficial to neurite outgrowth [16]. Here we see the same phenomenon; as the sensory neurons were treated with conditioned medium derived from human cells within the scaffold, any cell-cell interactions can be excluded in this experimental setting, and it must be soluble factors released by hSC that are influencing neurite outgrowth. We cannot rule out that in a coculture setup, contact-mediated support of neurite outgrowth is also important for neurite outgrowth. Nevertheless these current data show that hSCs seeded on the scaffold maintain their functionality regarding trophic factor secretion. Similar positive effects of human cells on neurite outgrowth were demonstrated with human dental pulp stem cells differentiated into Schwann cells which were used to manufacture an aligned tissue-engineered collagen construct [29]. In addition, also human umbilical cord Wharton's jelly-derived mesenchymal stem cells differentiated into Schwann cells had a positive effect on neurite outgrowth [30].

Since we used primary sensory neuron cultures that were inevitably associated with some satellite cells, we cannot rule out that at least a part of the effects induced by the soluble factors released by hSCs might be mediated via this cell type.

Future experiments are planned to demonstrate the cell-cell interactions of hSC within the scaffold in relation to direct, contact-mediated support of axonal regeneration.

## 5. Conclusion

Our data demonstrate that hSCs are adhered to and grow on Perimaix. Cells demonstrated a healthy morphology also in the center of the scaffold, which was confirmed by low levels of LDH release into the medium. As such, we can conclude that the scaffold is able to substantiate a beneficial environment to these cells.

We did not observe any differences in hSC behavior between the different prototypes suggesting that all four investigated prototypes were sufficiently capable of supporting hSC adherence and survival. Additionally, conditioned medium derived from hSCs grown on Perimaix was able to influence neurite outgrowth, indicating that hSC residing in a collagen matrix responded by releasing soluble factors that were beneficial to neurite growth. This may have implications for the clinical application of Perimaix, as it could also serve as a beneficial environment for nerve resident SC migrating from the nerve endings into the scaffold to support axonal regeneration.

## Figures and Tables

**Table 1:** Different prototypes of Perimaix used in this study.

Scaffold name	Abbreviation	Characterization
Scaffold 1	S1	Low degree of cross-linking/low collagen content
Scaffold 2	S2	Medium degree of cross-linking/low collagen content
Scaffold 3	S3	High degree of cross-linking/low collagen content
Scaffold 4	S4	High degree of cross-linking/high collagen content

## Abbreviations

ANT: Autologous nerve transplantation
DMEM: Dubecco's Modified Eagle Medium
DRG: Dorsal root ganglion
ECM: Extracellular matrix
FCS: Fetal calf serum
HBSS: Hank's Balanced Salt Solution
hSCs: Human Schwann cells
LDH: Lactate dehydrogenase
MACS: Magnetic antibody-linked cell sorting
PNI: Peripheral nerve injury
rSC: Rat Schwann cells
SC: Schwann cells
SHG: Second harmonic generation.
